# Ecological niche modeling of the pantropical orchid *Polystachya concreta* (Orchidaceae) and its response to climate change

**DOI:** 10.1038/s41598-020-71732-1

**Published:** 2020-09-09

**Authors:** Marta Kolanowska, Agnieszka Rewicz, Przemysław Baranow

**Affiliations:** 1 Department of Geobotany and Plant Ecology, Faculty of Biology and Environmental Protection, University of Lodz, Banacha 12/16, 90-237 Lodz, Poland; 2 Department of Biodiversity Research, Global Change Research Institute AS CR, Bělidla 4a, 603 00 Brno, Czech Republic; 3 Department of Plant Taxonomy and Nature Conservation, University of Gdańsk, Wita Stwosza 59, 80-308 Gdańsk, Poland

**Keywords:** Biogeography, Climate-change ecology, Plant ecology

## Abstract

Climate is the dominant control factor on the spatial distribution of organisms on a global scale and global warming is predicted to become a major cause of species extinctions. In our study ecological niche modeling (ENM) was used to estimate the effect of projected future climate changes on the pantropical orchid *Polystacha concreta* as well as to reconstruct changes in the distribution of the suitable climatic niches of this species since the Last Glacial Maximum (LGM). The study revealed small differences in the niches occupied by populations of *P. concreta* recorded in various continents; however, these alterations will become more significant in regard to future climatic change. While losses of suitable habitats of the studied orchid will occur in the Americas and Africa, global warming will be favorable for Asian populations. Our study suggests a significant loss of niches since the LGM which indicates that the currently observed loss of habitats is not only the result of human activity but also of natural changes of the Earth’s climate. From the obtained models we identified the areas that will be the most resistant regarding the modifications caused by climate change.

## Introduction

While the orchid family is one of the largest groups of flowering plants^1,2^, many members of this taxon are also among the most endangered – in part due to their dependance on particular climatic and vegetational niches, but also regarding their specific associations with mycorrhizal fungi and pollinators. Symbiotic mycorrhizal associations with fungal endophytes are indispensable for all orchids, especially in their early stages of development. Environmental factors greatly affect the functioning the orchid mycorrhiza. However, it is not clear to which extent fungal mycorrhiza in orchid germination and growth will remain functional with rising temperature, erratic rainfall, and reduced moisture^3^. Apart from mycorrhizal associations, highly specialized pollination mechanisms appear to play an important role in the rarity of orchids. Global warming has been shown to significantly impact the phenology of plants and their pollinators. Changes in species distribution caused by climate warming affect their interactions with other organisms. Matching of orchid flowering periods with the activity of the pollinating insects is important for successful cross-pollination^3^. Thus, for the effective conservation of orchids, all aspect of their associations with pollinators, mycorrhizal fungi, and host plants (regarding epiphytes) should be considered to determine in which way the complex biology of these plants will be affected by future global climate change^4^.

Undoubtedly, global warming affects all the above aspects of life strategies of orchids. Climate is the principal factor determining the spatial distribution of the main vegetational types on a global scale, while on a smaller scale, the impact of secondary factors such as type of the soil or topography are critical as well^5^. Undeniably, the currently rate of climate change is significantly affecting the distribution patterns of organisms and biodiversity in general^6,7^. Moreover, recent research has indicated that the future decrease of the ranges of some organisms will be accompanied by severe losses of cryptic evolutionary lineages^8^.

According to Fay^4^, as long as climate change does not make conditions unsuitable for species, conserving the habitats where orchids, and for epiphytes, their host trees grow should be treated as the highest priority; some countries have indeed established reserves especially for orchids. However, little is known about the possible impact of global warming on the occurrence of these rare plants. So far the evaluation of the impact of climate change on suitable habitat distribution has been conducted only for four invasive species^9–12^, several holomycotrophic orchids^13^, selected European *Dactylorhiza* Neck. *ex* Nevski representatives^14^, the small Neotropical genus *Diodonopsis* Pridgeon & M.W. Chase^15^, the Texas endemic *Spiranthes parksii* Correll^16^, and *Neottia cordata* (L.) Rich^17^. Additionally, rather regional studies have been published on orchids of New Guinea^18^ and species from Colombian dry forests^19^. Undoubtedly, effective conservation strategies should be supported by the recognition of suitable habitat distribution^20,21^. To ensure the success of long-term conservation, environmental protection efforts should be focused on those areas that will not be significantly affected by global warming within a predictable period of time.

In this study we applied an ecological niche modeling (ENM) approach not only to estimate the possible effect of global warming on the orchid *Polystacha concreta* (Hook.) Garay & H. R. Sweet, but also to reconstruct changes in the distribution of suitable climatic niches of this species since the Last Glacial Maximum (LGM). Moreover, we conducted statistical analyses to evaluate the difference in the niches occupied by populations of the studied orchid in various parts of its geographical range.

## Results

The niche overlap statistics calculated for models created using “fade-by-clamping” feature and without this function received high scores of both Schoener’s D (0.83–0.93) and Hellinger’s-based I (0.96–0.99) tests (Supplementary Annex 1). Also the visualization of binary models obtained using both approaches showed their similarity (Supplementary Annex 1). Here we discuss only results of analyses conducted without “fade-by-clamping” feature.

### ENM and changes in the distribution of suitable niches

The average training AUC for the replicate runs for all models received high scores (Table 1), which indicates that the MaxEnt models are reliable. The results of the True Skill Statistic (TSS) tests (Table 2) ranged between 0.49 and 0.93.

**Table 1:** The average training AUC for the replicate runs for the created models.

Region	LGM	Present	rcp2.6	rcp4.5	rcp6.0	rcp8.5
America	0.914 (SD = 0.007)	0.909 (SD = 0.008)	0.912 (SD = 0.009)	0.908 (SD = 0.009)	0.910 (SD = 0.009)	0.912 (SD = 0.009)
Africa	0.957 (SD = 0.004)	0.957 (SD = 0.004)	0.959 (SD = 0.004)	0.957 (SD = 0.004)	0.959 (SD = 0.004)	0.959 (SD = 0.004)
Asia	0.984 (SD = 0.003)	0.984 (SD = 0.003)	0.983 (SD = 0.003)	0.985 (SD = 0.003)	0.985 (SD = 0.003)	0.984 (SD = 0.003)

**Table 2:** Results of the TSS tests.

Region	LGM	Present	rcp2.6	rcp4.5	rcp6.0	rcp8.5
America	0.559	0.5113	0.577	0.585	0.530	0.489
Africa	0.803	0.761	0.777	0.7550	0.785	0.808
Asia	0.899	0.904	0.850	0.907	0.892	0.932

**Figure 1:**
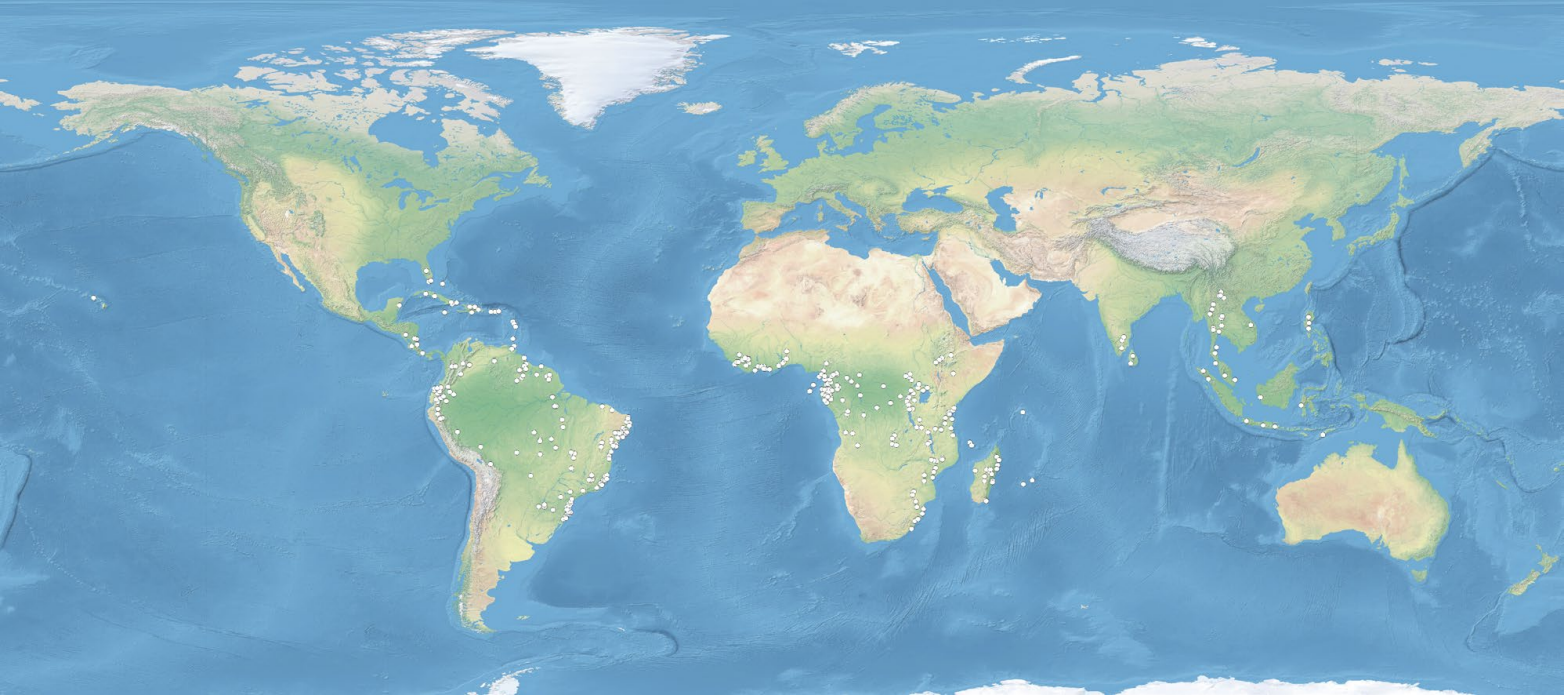
Locations of *P. concreta* used in ENM analysis. Map prepared in ArcGIS 10.6 (Esri, Redlands, CA, USA). Base map: Natural Earth II (https://www.shadedrelief.com/natural2).

Models created for the “present” time (1960–1990; Fig. 1) is consistent with the known geographical range of *P. concreta.* The only exception is southern Papua-New Guinea, northwestern Australia, New Caledonia, and Fiji which were indicated by ENM as areas suitable for the occurrence of the studied orchid and where so far this plant has not been found. On the other hand, the lowlands of Brazil, where *P. concreta* occurs, received low suitability score in our analyses.

**Figure 2:**
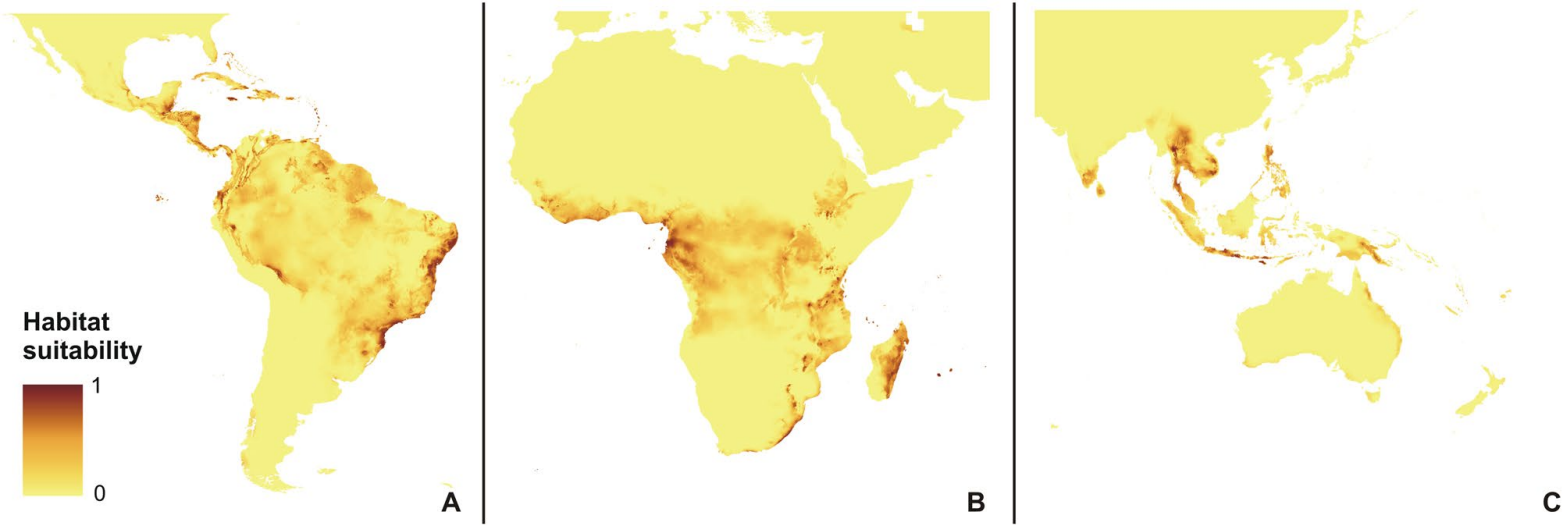
Current potential distribution of *P. concreta* in the Americas (**A**), Africa (**B**), and Asia (**C**). MaxEnt output maps visualized in ArcGIS 10.6 (Esri, Redlands, CA, USA).

Noticeably, the geographical range of potentially available habitats of the orchid was distinctly wider during the LGM than it is currently observed (Fig. 2). In the Neotropics the additional habitats were available for this species in the northeastern part of the Darién Gap, the eastern slope of the Andes, as well as in the extensive areas in the Amazon and Orinoco basins. In Africa the niches of the studied orchid were located also further from the coast when compared with the current distribution of *P. concreta.* Moreover, the areas within the Congo basin were much more suitable for this species. However, habitats in Madagascar were less suitable than at the present time. The emergence of Sundaland and the land connection between Papua-New Guinea and Australia significantly increased the coverage of areas where *P. concreta* was able to grow during the LGM.

**Figure 3:**
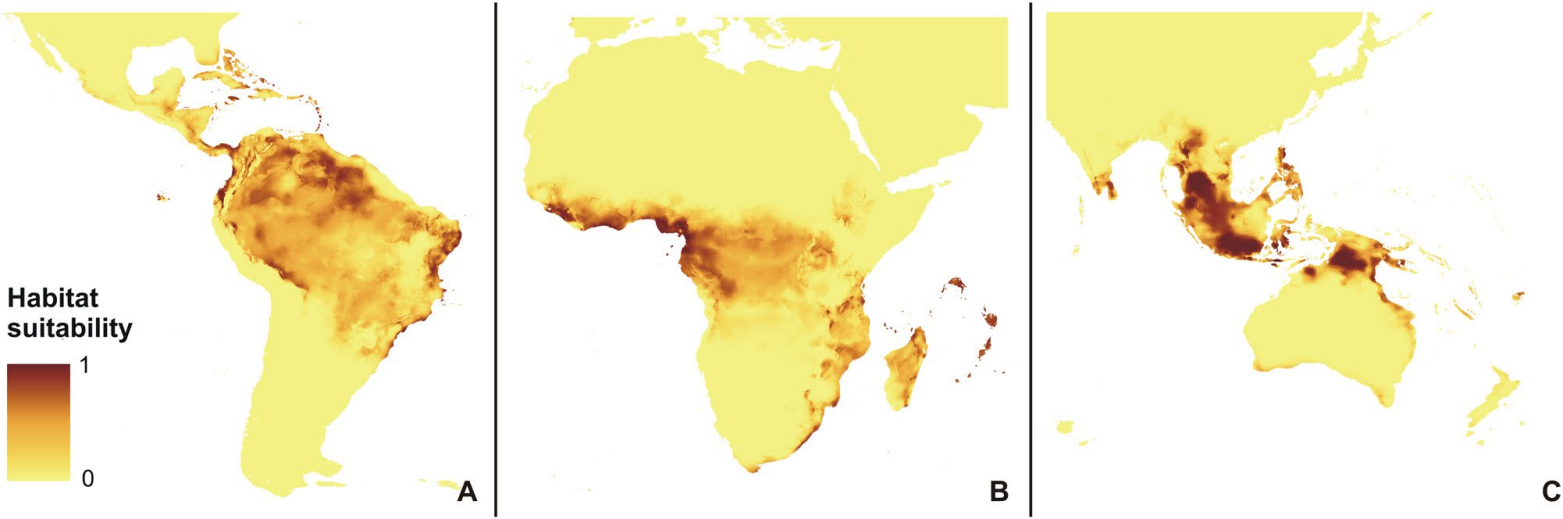
Glacial distribution of *P. concreta* in the Americas (**A**), Africa (**B**), and Asia (**C**). MaxEnt output maps visualized in ArcGIS 10.6 (Esri, Redlands, CA, USA).

**Figure 4:**
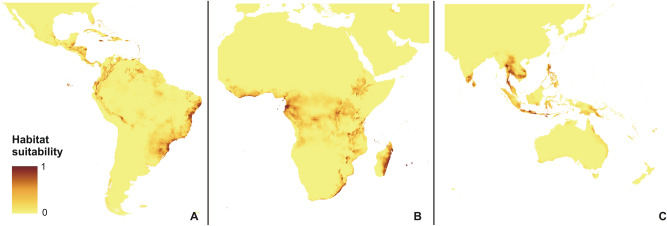
Future potential distribution of *P. concreta* in 2070 in the Americas (**A**), Africa (**B**), and Asia (**C**) based on rcp2.6 scenario. MaxEnt output maps visualized in ArcGIS 10.6 (Esri, Redlands, CA, USA).

**Figure 5:**
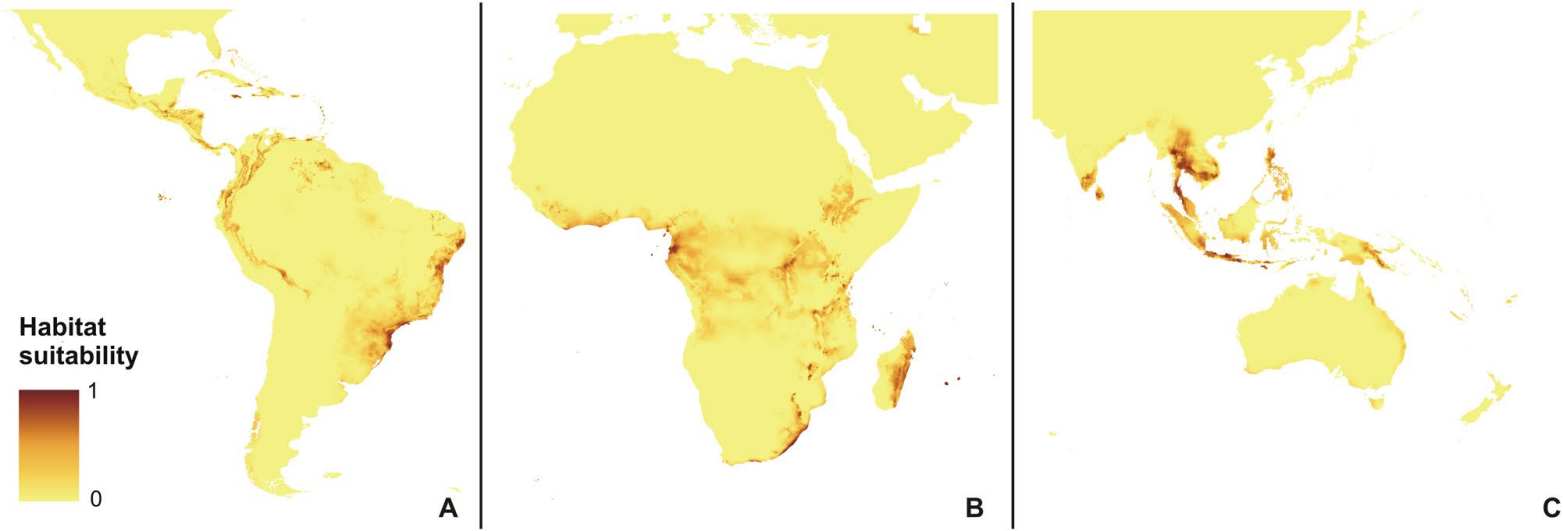
Future potential distribution of *P. concreta* in 2070 in the Americas (**A**), Africa (**B**), and Asia (**C**) based on rcp4.5 scenario. MaxEnt output maps visualized in ArcGIS 10.6 (Esri, Redlands, CA, USA).

**Figure 6:**
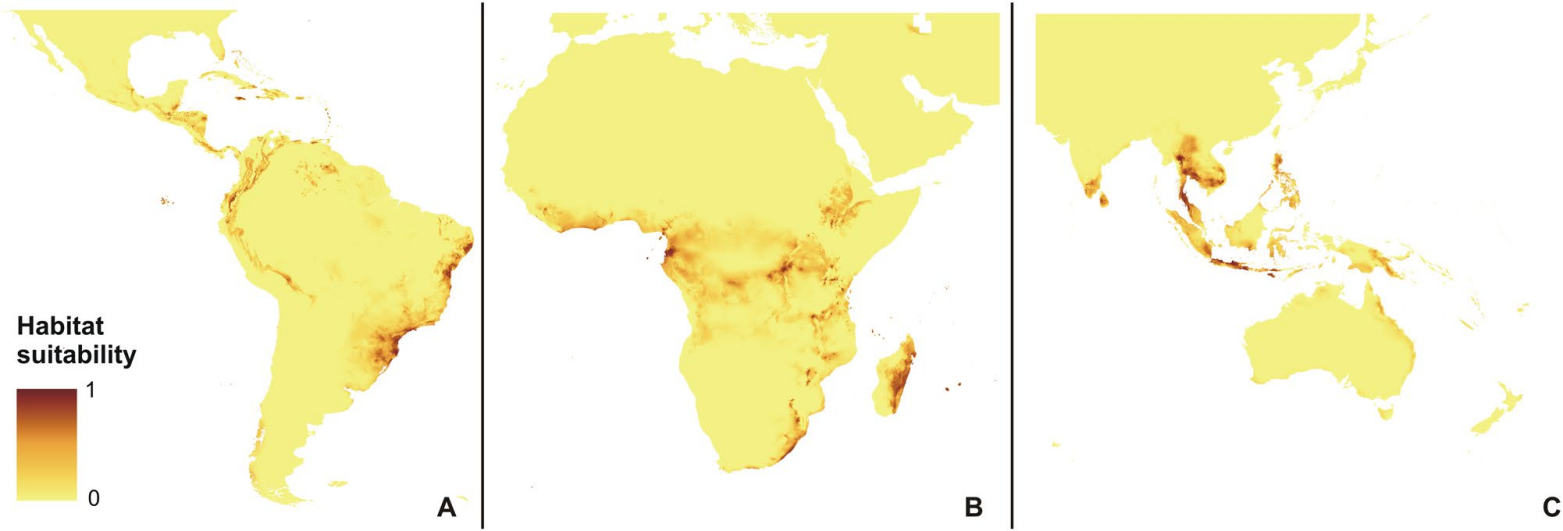
Future potential distribution of *P. concreta* in 2070 in the Americas (**A**), Africa (**B**), and Asia (**C**) based on rcp6.0 scenario. MaxEnt output maps visualized in ArcGIS 10.6 (Esri, Redlands, CA, USA).

**Figure 7:**
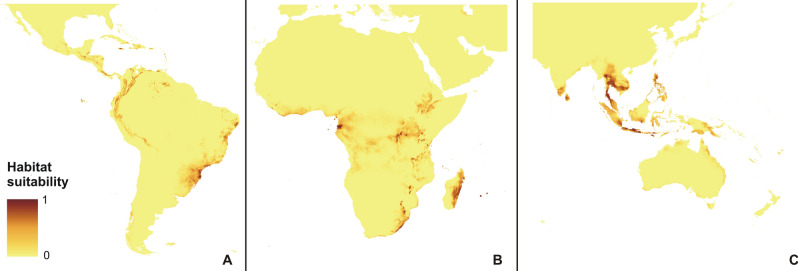
Future potential distribution of *P. concreta* in 2070 in the Americas (**A**), Africa (**B**), and Asia (**C**) based on rcp8.5 scenario. MaxEnt output maps visualized in ArcGIS 10.6 (Esri, Redlands, CA, USA).

**Figure 8:**
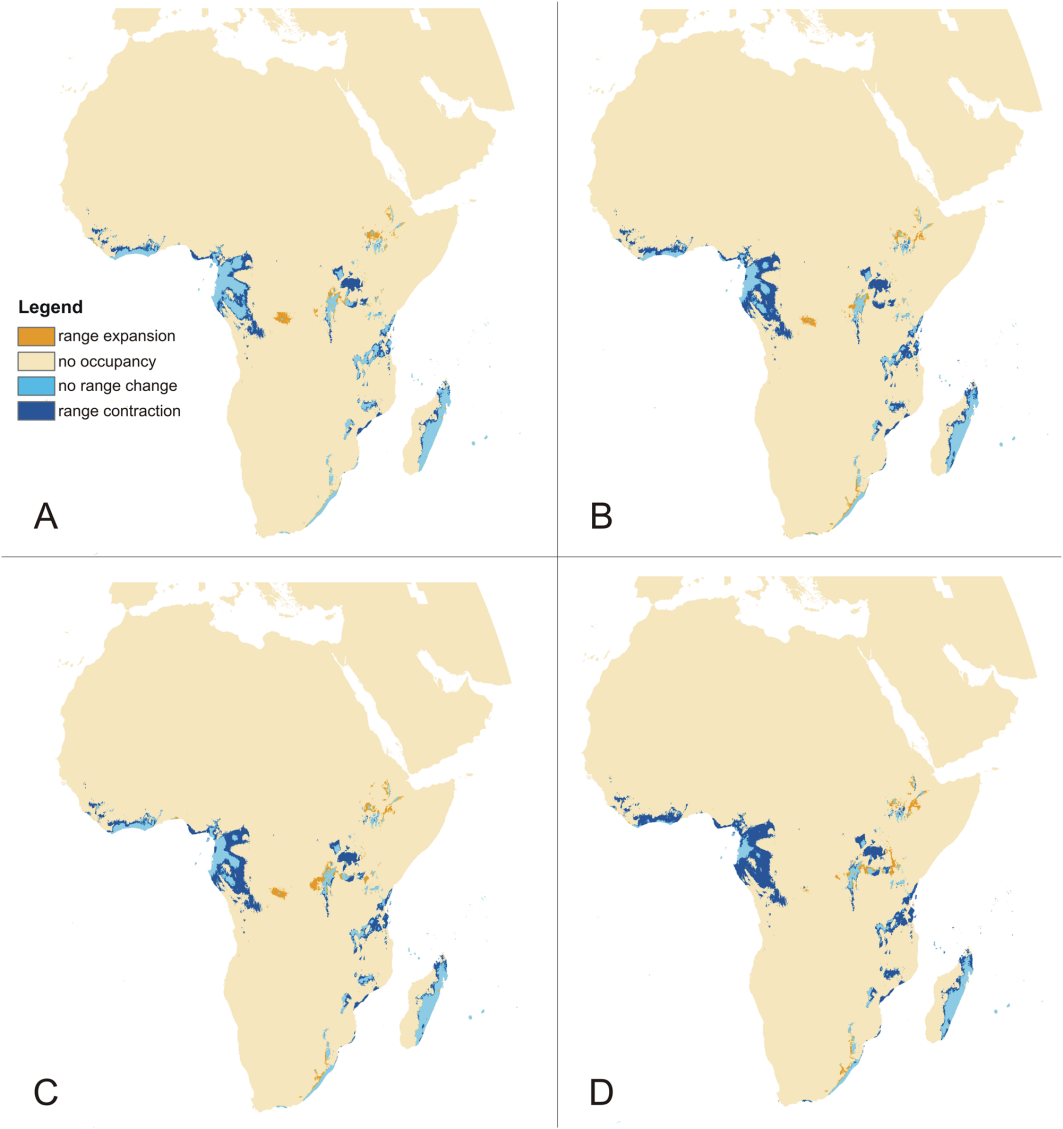
Changes in the distribution of suitable niches of *P. concreta* in Africa based on rcp2.6 (**A**), rcp4.5 (**B**), rcp6.0 (**C**), and rcp8.5 (**D**) scenarios. SDMtoolbox output maps visualized in ArcGIS 10.6 (Esri, Redlands, CA, USA).

**Figure 9:**
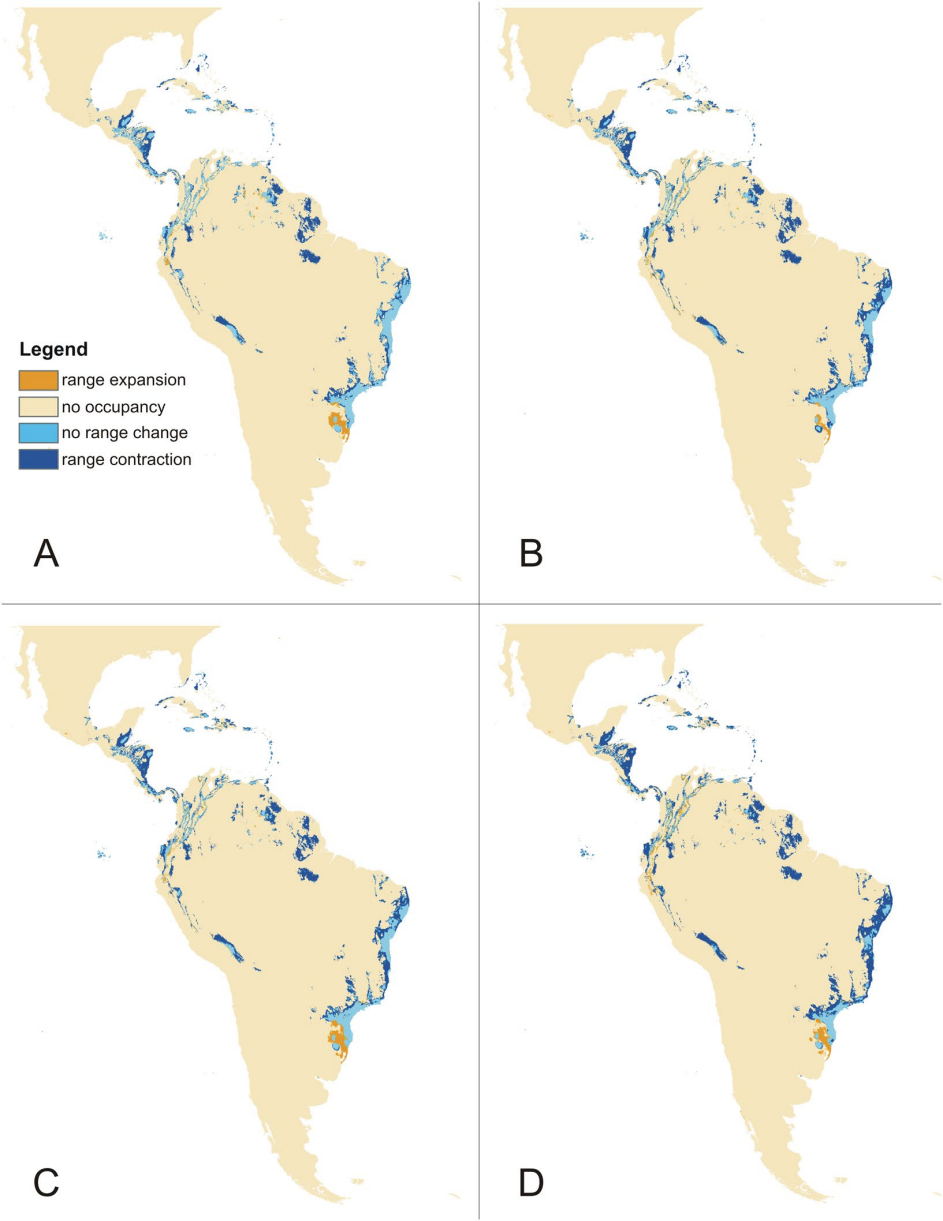
Changes in the distribution of suitable niches of *P. concreta* in the Americas based on rcp2.6 (**A**), rcp4.5 (**B**), rcp6.0 (**C**), and rcp8.5 (**D**) scenarios. SDMtoolbox output maps visualized in ArcGIS 10.6 (Esri, Redlands, CA, USA).

The rcp2.6 scenario of climate change would negatively affect the studied orchid in Africa and America (Fig. 3). Within the Neotropics, most inland lowland areas will no longer be suitable for *P. concreta.* In Africa, the coverage of currently existing niches will decrease. On the other hand, some additional habitats will be potentially available in Asia for this species in the Yunnan Plateau (Fig. 3). In America, rcp4.5 (Fig. 4), rcp6.0 (Fig. 5), and rcp8.5 (Fig. 6) will significantly reduce the coverage of niches suitable for *P. concreta* causing the highest losses in the inland lowland regions, decrease of habitats within the Andean mountains, and significantly limit the areas available for this species in Central America as well as on the Caribbean islands. Surprisingly, in the rcp4.5 scenario while coastal niches of the studied orchid are projected to become more limited, the additional habitats would be available in the eastern and southern parts of the Congo basin (Fig. 4). The later will not be suitable for *P. concreta* in any other climate change scenario. In the worst-case scenario of rcp8.5, the studied orchid is predicted to become extinct over the entire continent (Fig. 6). Unlike in the Neotropics and Africa, climate change in Asia will expand the coverage of existing suitable niches, especially in the insular region, but also in south and eastern India and the Indochina Peninsula and montane regions of Thailand and Laos (Figs. 3, 4, 5, 6). Moreover, in the rcp8.5 scenario (Fig. 6) a significant habitat extension is expected for the Philippines and southern Borneo. The changes in distribution of highly suitable areas (≥ 0.4) are presented in Figs. 7, 8 and 9.

### Limiting factors and variation of occupied niches

The estimates of relative contributions of the environmental variables to the Maxent models are provided in Table 3. The distribution of American populations is limited by the mean diurnal range (bio2). The Asian range is affected mostly by the temperature seasonality (bio4), while the variable most influencing the African model is annual precipitation (bio12). Somewhat less important factors determining the occurrence of *P. concreta* in the Americas are temperature seasonality (bio4) and annual mean temperature (bio1). The distribution of this species in Asia is additionally limited by the isothermality (bio3) and by the precipitation seasonality (bio15). African populations depend also on the precipitation within the driest month (bio14) and by the precipitation of the warmest quarter (bio18).

**Table 3:** Estimates of relative contributions of the environmental variables to the Maxent models.

Region	var. 1	var. 2	var. 3
America	bio2 (20.1%)	bio4 (20.0%)	bio1 (15.6%)
Africa	bio12 (39.3%)	bio18 (20.7%)	bio14 (16.7%)
Asia	bio4 (36.7%)	bio3 (14.5%)	bio15 (11.0%)

**Figure 10:**
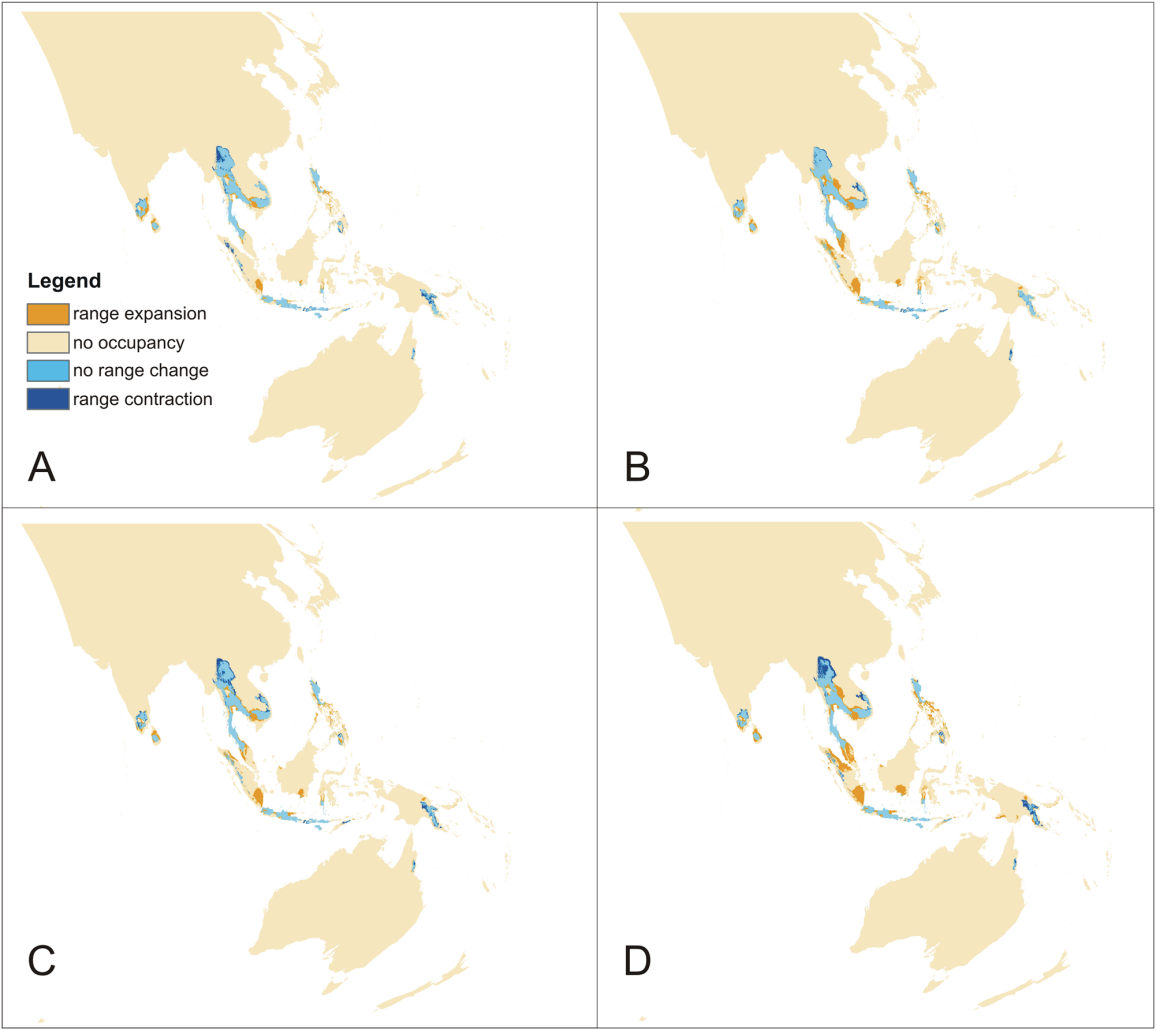
Changes in the distribution of suitable niches of *P. concreta* in Asia based on rcp2.6 (**A**), rcp4.5 (**B**), rcp6.0 (**C**), and rcp8.5 (**D**) scenarios. SDMtoolbox output maps visualized in ArcGIS 10.6 (Esri, Redlands, CA, USA).

**Figure 11:**
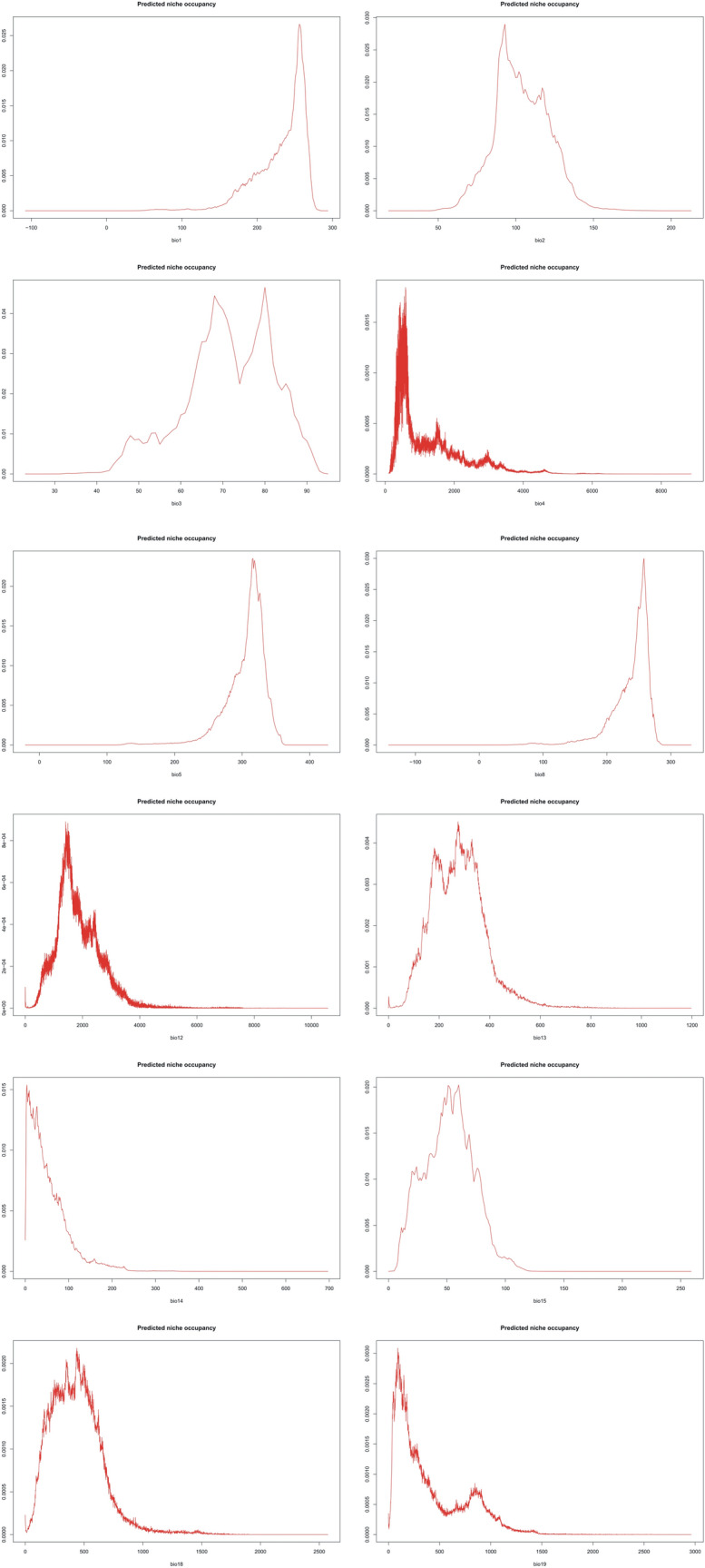
PNO profiles of American *P. concreta.*

**Figure 12:**
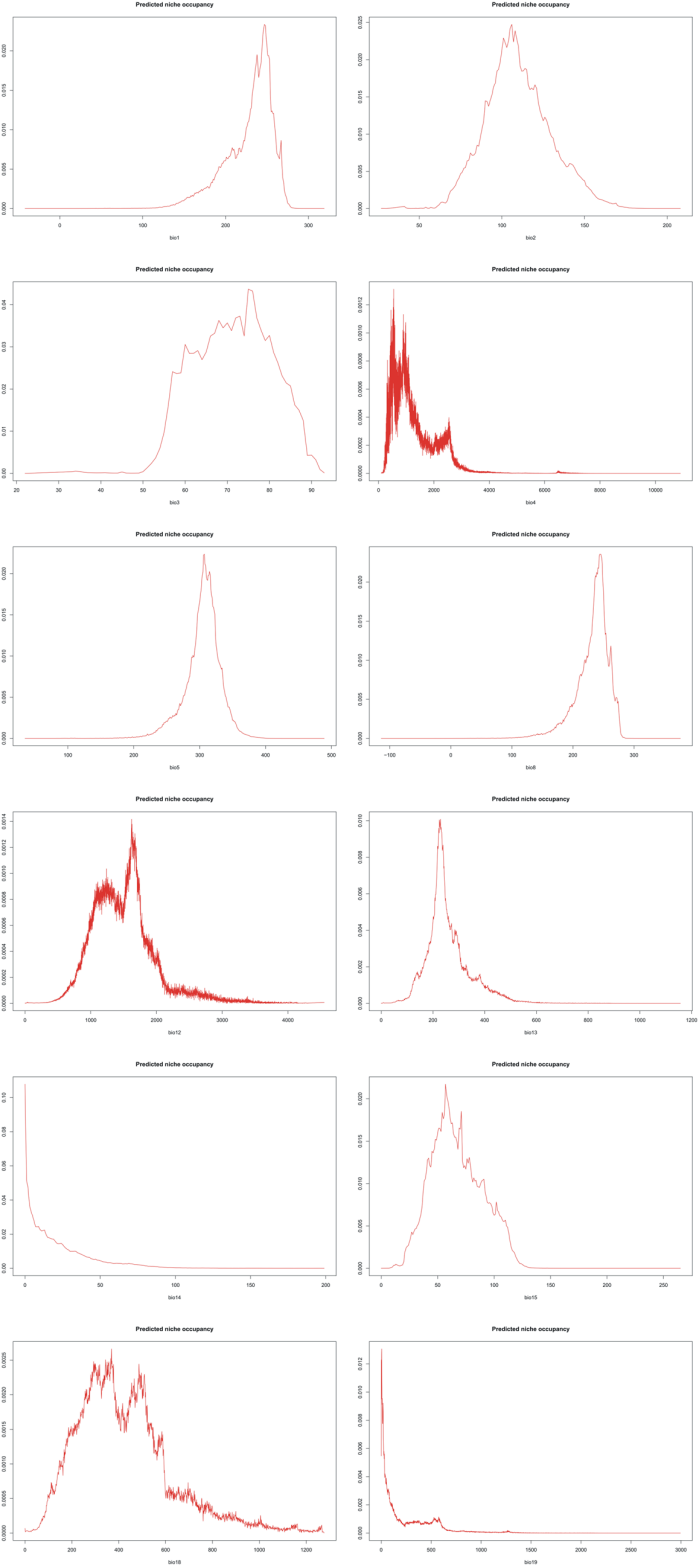
PNO profiles of African *P. concreta.*

**Figure 13:**
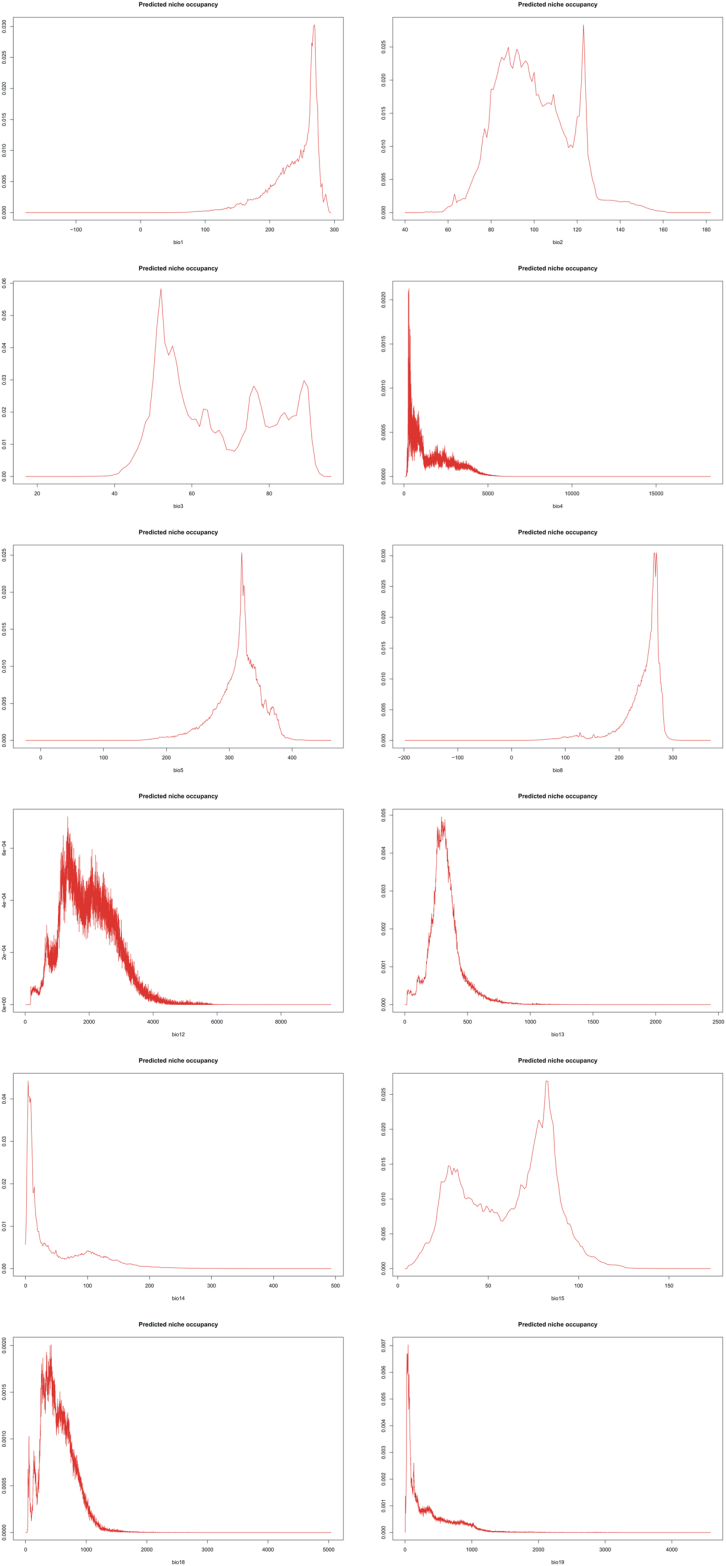
PNO profiles of Asian *P. concreta.*

The PNO profiles of all three geographical groups of *P. concreta* are presented in Figs. 10, 11, 12 and 13. The differences in the tolerance for various climatic factors can be easily recognized in respect to several variables. In case of bio2, two distinct peaks of high suitability of this variable are observed only within Asian populations. On the other hand, for bio3 the values of 60–80 African populations are relatively stable. In respect to the same variable, three peaks of higher suitability in Asian populations and two peaks in the Neotropical ones were recognized. Also, two distinct ranges of bio15 values are suitable for Asian populations. In respect to bio18, two peaks of ca. 270–380 and 430–530 were recognized as the most appropriate for the occurrence of African populations.

**Figure 14:**
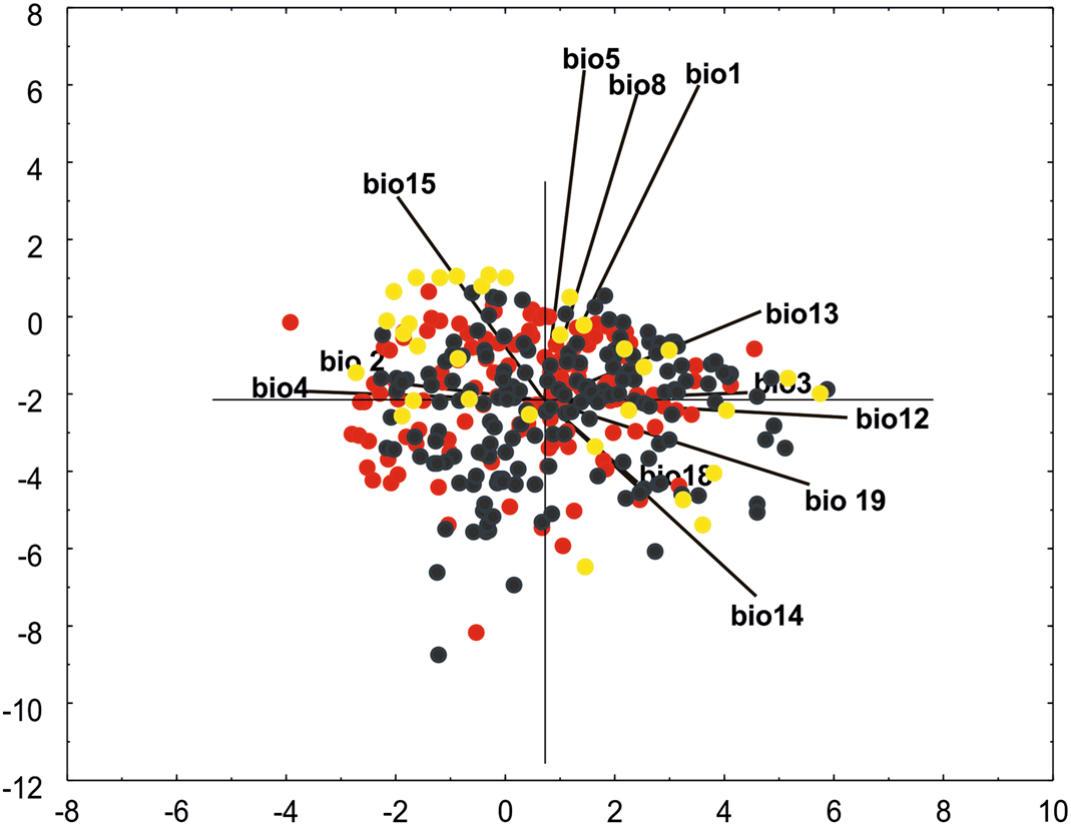
Ordination diagrams of the Principal Component Analysis (PCA) of *P. concreta* based on 12 bioclimatic variables. Explanation: (Asia: yellow dots, the Americas: black dots, Africa: red dots).

In the PCA analysis the first component accounted for 27.4% of the total variance and the second component accounted for 25.1% of the total variance*.* A very small distinct group was distinguished for populations from America, which was related with bio12; this could be related to the fact that a large group of American populations had higher annual precipitation values. Part of the Asian population was related to bio15, which could have resulted from high levels of precipitation seasonality. Similarly, two overlapping groups from Africa and Asia populations were correlated with bio5 (Fig. 14).

### Coverage of suitable niches

To evaluate changes in the coverage of suitable niches we calculated the number of grid cells with values of at least 0.4. The areas of range changes were calculated using SDMToolbox 2.4. In most parts of the geographical range of *P. concreta* the area suitable for the distribution of this species is currently decreasing. The current coverage of suitable habitats constitutes a small portion of the glacial potential range of this species – 18.99% in Asia, 25.83% in the Americas, and 38.15% in Africa (Table 4). The response to the anticipated future climate change varies between continents. In Africa and the Americas the most damaging effects are projected in the rcp8.5 scenario (Table 5). In this case the total area potentially available for *P. concreta* will be limited to 617,861.57 km^2^ in Africa and to 566,244.72 km^2^ in the Americas*.* In Asia the highest habitat loss is projected in the rcp2.6 scenario. However, in all examined scenarios also a significant range expansion of suitable niches in this region (216,754.03–653,555.51 km^2^) would occur (Table 5).

**Table 4:** Changes in the suitable niches coverage since LGM.

Region	Range expansion (km^2^)	No occurrence (km^2^)	No change (km^2^)	Range contraction (km^2^)	Total habitat loss (km^2^)	% of habitat loss
Africa	310,173.95	33,231,743.051	1,241,291.69	2,462,528.41	− 2,152,354.46	61.85%
America	689,171.13	15,224,781.06	1,080,186.07	5,554,847.18	− 4,865,676.04	74.17%
Asia	176,927.18	29,901,328.99	896,327.55	1,648,967.93	− 1,472,040.74	81.01%

**Table 5:** Changes in the coverage of suitable niches of *P. concreta*.

Region	Scenario	Grid cells ≥ 0.4	Range expansion (km^2^)	No occurrence (km^2^)	No change (km^2^)	Range contraction (km^2^)	Habitat loss/expansion (%)
Africa	Present	73,623	–	–	–	–	
	rcp26	51,804	127,480.57	35,569,010.09	959,659.09	591,806.55	− 29.93
	rcp45	38,988	121,439.42	35,575,051.24	688,958.09	862,507.55	− 47.77
	rcp60	43,934	168,474.08	35,528,016.58	748,547.6	802,917.99	− 40.89
	rcp85	29,844	131,610.74	35,564,879.92	486,250.83	1,065,214.81	− 60.18
America	Present	85,191	–	–	–	–	
	rcp26	49,039	130,510.86	20,649,117.38	874,708.55	894,648.65	− 43.19
	rcp45	36,635	97,900.02	20,681,728.22	652,959.38	1,116,397.82	− 57.56
	rcp60	39,727	160,353.48	20,619,274.75	645,937.58	1,123,419.62	− 54.43
	rcp85	27,871	164,584.56	20,615,043.67	401,660.15	1,367,697.05	− 68.00
Asia	Present	51,347	–	–	–	–	
	rcp26	54,082	216,754.03	31,333,542.89	917,593.63	155,661.10	+ 5.69
	rcp45	67,976	455,291.68	31,095,005.25	975,181.41	98,073.32	+ 33.28
	rcp60	59,113	363,899.29	31,186,397.63	881,130.77	192,123.96	+ 16.01
	rcp85	70,852	653,555.51	30,896,741.41	843,915.14	229,339.60	+ 39.53

## Discussion

In the present study we applied the ENM approach to estimate possible changes in the distribution of the suitable niches of a pantropical orchid species, *P. concreta.* This method has become one of the most important tools for the assessment of the impact of climatic change and for the detection of failures in nature management plans^22^. Mapping the future distribution of rare plants allows to appropriately prioritize conservation areas^23–25^. However, in our analyses we additionally evaluated the long-term modifications in the location and coverage of the suitable niches of the studied orchid by comparing current ranges of this species with projected potential ranges during the LGM. We consider that element important to uncover the general patters of the species' conditions at a global scale.

Our study results indicated small differences in the niches occupied by populations of *P. concreta* in various continents and the variance in the climatic factors crucial for the occurrence of this species within its geographical range. These alterations combined with different bioclimatic conditions characterized for each continent are, however, very significant when anticipated future climatic change is considered. While we expect to observe the loss of suitable habitats of the studied orchid in the Americas and Africa, global warming will be favorable for Asian populations.

Another interesting result of our analyses is the significant loss of niches since the LGM which indicates that the currently observed loss of habitats is not only the result of human activity but is also driven by the natural changes of the Earth’s climate. A similar pattern was recently described for the Neotropical orchid genus *Diodonopsis*^14^. The most reduced coverage of suitable habitats of *P. concreta* is currently observed in Asia; this is easily explained, however, by the increase of the sea level within the Sahul and the Sunda Shelfs. At the same time, the existing Asian populations of *P. concreta* will most likely not be negatively affected by future global warming due to the emergence of additional suitable niches in numerous inland and insular areas. On the other hand, in Africa where after the LGM 48% of suitable niches remained available for the studied orchid, the species can go completely extinct in the rcp8.5 climate change scenario. In America, where a significant loss of niches was observed since the LGM, global warming will cause a serious decrease in the coverage of suitable habitats, but the species should be able to survive in the remaining refugia.

The obtained models allow to indicate the areas which are potentially the most resistant to the modifications caused by climate change and thus may be able to serve as refuges for the studied species in the future. Since global warming can currently be seen as one of the most serious threats for species richness, habitat stability should be taken into consideration in establishing nature conservation areas. In the case of *P. concreta* there are several regions where long-term conservation efforts will be more effective considering the predicted significant climate change projected in the rcp8.5 scenario. In the New World such “resistant” areas, where despite global warming the niche will be suitable for the studied species in the future, are found in Central America (especially in Jamaica), the Andes, Eastern Venezuela, and the coastal part of Eastern Brazil. The results of predicted changes are interesting in Asia as all the modelled scenarios indicated that Asiatic habitats of *P. concreta* will be resistant to global warming and will even increase their coverage. In Africa climate change will substantially affect present habitats of *P. concreta*. In this region special attention on biodiversity conservation should be focused in eastern Madagascar and the eastern coast of the Gulf of Guinea where suitable niches of the studied orchids are predicted to exists in the future.

Studies on historical biogeography of orchids are very limited due to very scarce fossil material representing Orchidaceae and/or their ancestors^26,27^. So far no paleobotanic trace of any member of *Polystachya* has been revealed. For that reason our analysis of the glacial distribution of this species may be somewhat biased. While orchids are usually considered to be highly specialized with a rather narrow ecological tolerance^28,29^, we are not able to exclude the possibility that niche shift occurred in the life history of *P. concreta* and that the climatic preferences of this species changed over time. On the other hand, we believe that the projections of future modification of the potential range based on climate characteristics of the studied orchid are precise. Even if *P. concreta* could potentially adapt to somewhat different climate conditions it is unlikely to happen on a broad scale over a short period of time (by 2070) as by our analysis.

Considering the complex life strategy of *P. concreta,* this study has some limitations. According to information provided on herbarium labels *P. concreta* grows epiphytically on various tree species and its occurrence is not related with any particular phorophyte, thus we could not include this factor into our analyses. The species was recorded on coffee trees in Indonesia (*Vermeulen J.J. 2279*). *Coffea* plants were introduced to this country in 1699^30^, indicating that *P. concreta* adapted to this phorophyte relatively recently. Occasionally, the specimens of the studied orchid were also found growing as terrestrials or lithophytic herbs. Thus, we assume that neither the substrate nor the tree host consistute factors limiting the occurrence of *P. concreta*.

While symbiotic fungi are vital for the germination of orchid seeds, the specificity of members of Orchidaceae for their mycorrhizal partners and the effects of the fungi on orchid growth are not perfectly clear. Numerous studies have indicated that Orchidaceae are able to form associations with various fungi (see, for instance^31–33^). Due to the lack of comprehensive data on myccorhizal symbionts of *P. concreta* we were not able to include modifications of the availability of fungal components in our analyses. Senthilkumar^34^ reported associations of this orchid with worldwide distributed *Rhizoctonia*, while as recently as 2003 a completely new species of Tulasnellaceae was isolated from the roots of *P. concreta*^35,36^. According to a recent analysis on the relationship of the endangered terrestrial orchid *Liparis loeselii*^37^, mycorrhizal communities associated with this orchid vary among geographical locations and plant life cycle stages, but the observed variations did not affect seed germination. In our opinion, when considering the broad range of habitats which can be inhabited by *P. concreta* it would seem that the mycorrhizal specificity is not a factor limiting the geographical distribution of this species.

With the broad geographical range of *P. concreta* little is known about its pollination biology and thus we were not able to include the potential modification of the geographical ranges of the pollinators of the studied species in our analyses. The plants are autogamous in the West Indies^38^, but cross-pollination was reported from other areas. This orchid is undoubtedly pollinated by halictid bees, such as *Dialictus creberrimus*, which visit the flower to collect lip hairs known as pseudopollen^39^. Moreover, certain Neotropical Meliponini bees (*Plebeia droryana*, *Tetragonisca angustula*, and *Trigona spinipes*) were reported to transfer pollen. Pansarin et al.^34^ included also the Tetrapediini member *Paratetrapedia* aff. *fervida* as a pollinator of *P. concreta* in Brazil*.*

In our analyses of the potential distribution of *P. concreta* we included exclusively climatic variables. This approach was motivated by the lack of comprehensive data on pollination ecology, mycorrhizal specificity, and deficiency of information about preferred phorophytes of the studied species. However, considering the high tolerance of this orchid for their substrate, host trees, and the possibility of self-pollination and records of various pollinators from different geographical locations, we assume that only climatic factors can limit the general distribution of *P. concreta.* Thus, the species seems to be an appropriate model for studies focused on the impact of global climate changes on pantropically distributed herbs.

Some recent studies have focused on the potential migration of various plant species to non-analogous climate conditions^40^. It is hypothetically possible that current realized niche of *P. concreta* constitutes just a small part of its fundamental niche. While numerous herbs and trees are known to adjust physiologically and morphologically to shifting environmental conditions^41–43^, orchids are generally regarded as plants with an insignificant potential of adapting to different niches^11^. The first substantial shift of climatic preferences of an orchid was reported in 2018 for the invasive *Disa bracteata*^44^; and in this case, comprehensive and accurate documentation of the introduction of this orchid was available to the authors. The lack of data on the migration (e.g. time since introduction to various continents) and dispersal ability of *P. concreta* offset the possibility of evaluating the potential of this orchid for niche expansion to new climate space^45^.

To conclude, despite the various limitations discussed above, our study revealed an interesting pattern of responses of pantropical orchid species to global warming. It should be particularly noted that in case of *P. concreta* a decrease of suitable niche coverage has been observed since the LGM suggesting that this habitat loss is not exclusively human-driven but at least partially a result of natural changes of climate. While a loss of niches of *P. concreta* will be observed in the Americas and Africa, global warming will be favorable for Asian populations. We were able to delimit areas where climate changes will not significantly affect the availability of suitable habitats of the studied orchid. In our opinion, for determining long-term conservation measures, habitat conservation efforts should be concentrated to these regions.

## Materials and methods

### Studied species

Most species of *Polystachya* Hook. occur in tropical Africa; only those belonging to the nominal section have achieved pantropical distribution with representatives in continental Africa, Madagascar, Central and South America, Florida, Asia, and Australasia^46^. Usually, individual species are restricted to one continent. The exception is *Polystacha concreta*, which is widely distributed throughout the Neotropical and tropical African forests to the Indian Ocean islands, southern India, Sri Lanka, and Southeast Asia^47,48^.

These are epiphytic, rarely lithophytic or terrestrial plants and they are found in tropical wet montane forest, submontane forest, riverine forest, as well as in savannas and along creeks. The altitudinal range of *P. concreta* extends from elevations near sea level up to about 2,400 m. The plants are highly variable in vegetative characters. They can grow as robust herbs with long, branched inflorescences composed of numerous flowers or as tiny plants with few-flowered, simple racemes^49^. Their elongate stems are basally swollen into ovoid pseudobulbs bearing 2–6, thin-textured, oblong-elliptic to elliptic, obtuse or acute leaves. The apical inflorescence can reach a height of 50 cm. The elongate, compressed peduncle is completely enclosed by scarious sheaths. The non-resupinate, fleshy, rather small flowers are yellow, yellowish green, or dull pink^50^.

### Niche modeling and statistical analyses

A database of *P. concreta* locations was created from the examination of herbarium material (Supplementary Annex 2) and information from the Global Biodiversity Information Facility (GBIF)^51^. The GBIF catalog was used exclusively to enrich the database of Neotropical locations of this species. In America *P. concreta* is rather easily distinguished from other members of the genus based on the prominent lip callus extending to about half of the lip's length. Undoubtedly, *P. concreta* is difficult to identify in the Old World where there are numerous members of the genus. For that reason, we did not use public repositories to gather African and Asian records of the studied species. Specimens collected in these areas were examined using stereoscopic microscope to ensure correct identification of the species. Duplicate records were removed from the analysis. The database included initially a total of 581 records (286 from America, 256 from Africa, and 39 from Asia). To reduce spatial autocorrelation of the samples, spatial thinning was conducted using SDMtoolbox 2.3 for ArcGIS. The data were rarified using 30-km resolution and by designating the minimal distance of 5 km on calculated climatic habitat heterogeneity. The final database included a total of 354 records: 32 from Asia, 169 from America, and 153 from Africa (Supplementary Annex 3).

The ecological niche modelling was conducted using the maximum entropy method in MaxEnt version 3.3.2^52–54^ based on presence-only observations of this species. Because some previous studies^55^ indicated that use of a restricted area in ENM analysis is more reliable than calculating habitat suitability on the global scale, the region of our analysis was restricted for each part of the known geographical range of *P. concreta.* The background for modeling of the Asian group extended from 51.20°N to 53.71°S and from 64.83°E to 180°E. Analysis of the American part of the range included the region between 34.25°N and 59.00°S and between 127.00°W and 29.87°W, while the African group included areas extending from 40.25°N to 52.29°S and from 23.83°W to 64.12°E. Of 19 climatic variables (“bioclims”, Table 6) in 2.5 arc minutes (± 21.62 km^2^ at the equator) of interpolated climate surface^56^ in WorldClim (version 1.4^57^) seven were removed as they were significantly correlated with one another (above 0.9) as evaluated by Pearsons’ correlation coefficient computed using ENMTools v1.3^58^. From each pair of correlated “bioclims” we removed the one which that, based on our experience, was less important for species distribution. The following variables were used in the analysis: bio1, bio2, bio3, bio4, bio5, bio8, bio12, bio13, bio14, bio15, bio18, and bio19 (Table 5). Because the altitudinal range of the species is very broad (ca. 2,400 m) and simultaneously the elevation is highly correlated with climatic data, the Digital Elevation Model (DEM) was not included in our analyses. Due to the variable habit of *P. concreta* which can grow as a terrestrial plant, but is also commonly found on trees, fences, and rocks, the soil data were not encompassed in modelling. Numerous locations of the studied species included in our input database are from before 1990, i.e., before significant anthropogenic climate change. However, because WorldClim 1.4 layers for the “present time” were created based on climate conditions recorded between 1960 and 1990, the created models should not be biased by the old records included in the analyses.

**Table 6:** Codes of climatic variables developed by Hijmans et al.^56^.

Code	Description
bio1	Annual mean temperature
bio2	Mean diurnal range = mean of monthly (max temp − min temp)
bio3	Isothermality (bio2/bio7) * 100
bio4	Temperature seasonality (standard deviation * 100)
bio5	Max temperature of warmest month
bio6	Min temperature of coldest month
bio7	Temperature annual range (bio5–bio6)
bio8	Mean temperature of wettest quarter
bio9	Mean temperature of driest quarter
bio10	Mean temperature of warmest quarter
bio11	Mean temperature of coldest quarter
bio12	Annual precipitation
bio13	Precipitation of wettest month
bio14	Precipitation of driest month
bio15	Precipitation seasonality (coefficient of variation)
bio16	Precipitation of wettest quarter
bio17	Precipitation of driest quarter
bio18	Precipitation of warmest quarter
bio19	Precipitation of coldest quarter

CCSM4 (Community Climate System Model 4) simulation was used to reconstruct the distribution of the studied species during the last glacial maximum (LGM). The environmental data for this modeling were provided by the Coupled Model Intercomparison Project (CMIP5) and downloaded from WorldClim (version 1.4^57^). This method has previously been applied in biogeographical studies on various organisms (see, e.g., Chung et al*.*^59^, Kim et al*.*^60^). Predictions on the future extent of climatic niches of *P. concreta* in 2070 were made using climate projections obtained from the Community Climate System Model (CCSM4). Four representative concentration pathways (RCPs: rcp2.6, rcp4.5, rcp6.0, rcp8.5) were analyzed. These four scenarios describe different potential future climates on the basis of different assumed amounts of emitted greenhouse gases. The RCPs are named after a possible range of radiative forcing values in 2100^61,62^, relative to pre-industrial values (+ 2.6, + 4.5, + 6.0, and+ 8.5 W/m^2^, respectively). These climate projections have been used in several previous studies on threatened plants and endangered animals^63,64^.

In all analyses the maximum number of iterations was set to 10,000 and convergence threshold to 0.00001. Background predictions were created. We applied the “random seed" option which provides a random test partition and background subset for each run; and 10% of the samples were used as test points. The run was performed as a bootstrap with 100 replicates, and the output was set to logistic. All operations on GIS data were carried out on ArcGis 10.6 (Esri, Redlands, CA, USA). The evaluation of the created models was made using the most common metrics – the area under the curve^65,66^ and the True Skill Statistic (TSS)^67^. The AUC was calculated in MaxEnt and the TSS value was derived using R^67,68^.

Separated analyses of the future distribution of suitable niches of *P. concreta* were also conducted using “fade-by-clamping” feature to remove heavily clustered pixels from the final predictions^69^. The overlap of created maps was visualized and the niche overlap between the models was calculated using Schoener’s D^70^ and Hellinger’s-based I^71^ statistics.

Predicted niche occupancy (PNO) profiles were plotted to visualize climatic preferences of *P. concreta*. PNO integrates species probability (suitability) distributions derived with MaxEnt with respect to a single climatic variable^72^.

The SDMtoolbox 2.3 for ArcGIS was used to visualize changes in the distribution of suitable niches of the studied orchid caused by global warming^73^. To compare the distribution model created for current climatic conditions with four future models all SDMs were converted into binary rasters and projected using Mollweide as Equal‐Area projection. Most known populations of *P. concreta* occur in areas for which the suitability calculated in the ENM analysis was 0.4. This value was therefore used as the threshold for binary rasters.

Principal Components Analysis (PCA) was used to assess variability within the geographic distribution of *P. concreta* and bioclimatic variables. The matrix data were log-transformed before performing principal component analysis. The software package Statistica v.13.3 was used for ordination analysis^74,75^.

## Supplementary information

Supplementary file 1.

Supplementary file 2.

Supplementary file 3.
