# Supplementary material for: Ecological niche modeling of the pantropical orchid Polystachya concreta (Orchidaceae) and its response to climate change
Source: Sci Rep. 2020 Sep 9;10:14801. doi: 10.1038/s41598-020-71732-1 (PMC7481249; doi:10.1038/s41598-020-71732-1)
Supplement: Supplementary file 1 [file 41598_2020_71732_MOESM1_ESM.doc]

***Ecological niche modeling of the pantropical orchid Polystachya concreta (Orchidaceae) and its response to climate change***

**Marta Kolanowska**^1,2^**, Agnieszka Rewicz**^1^ **& Przemysław Baranow**^3^

^1^University of Lodz, Faculty of Biology and Environmental Protection, Department of Geobotany and Plant Ecology, Banacha 12/16, 90-237 Lodz, Poland.

^2^Department of Biodiversity Research, Global Change Research Institute AS CR, Bělidla 4a, 603 00 Brno, Czech Republic.

^3^University of Gdańsk, Department of Plant Taxonomy and Nature Conservation, Wita Stwosza 59, 80-308 Gdańsk, Poland.

Correspondence and requests for materials should be addressed to A.R. (email: agnieszka.rewicz@biol.uni.lodz.pl)

**Annex 1. Results of analyses conducted using “fade-by-clamping” function in MaxEnt.**

Fig. 1. Overlap of African binary models created using “fade-by-clamping” option in MaxEnt (red hatch) and without this option (blue filling) based on rcp2.6 (A), rcp4.5 (B), rcp6.0 (C), and rcp8.5 (D) scenarios.


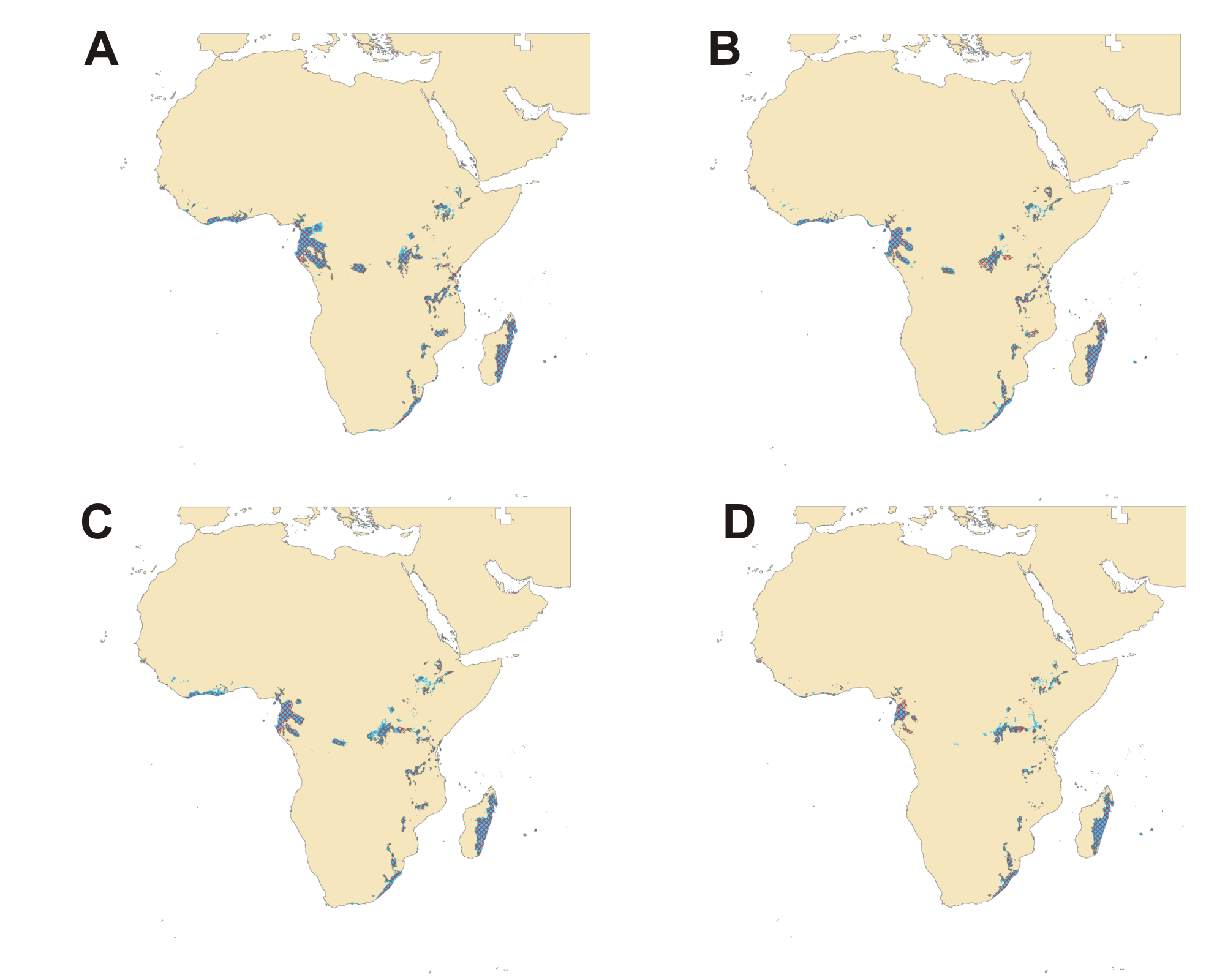


Fig. 2. Overlap of American binary models created using “fade-by-clamping” option in MaxEnt (red hatch) and without this option (blue filling) based on rcp2.6 (A), rcp4.5 (B), rcp6.0 (C), and rcp8.5 (D) scenarios.


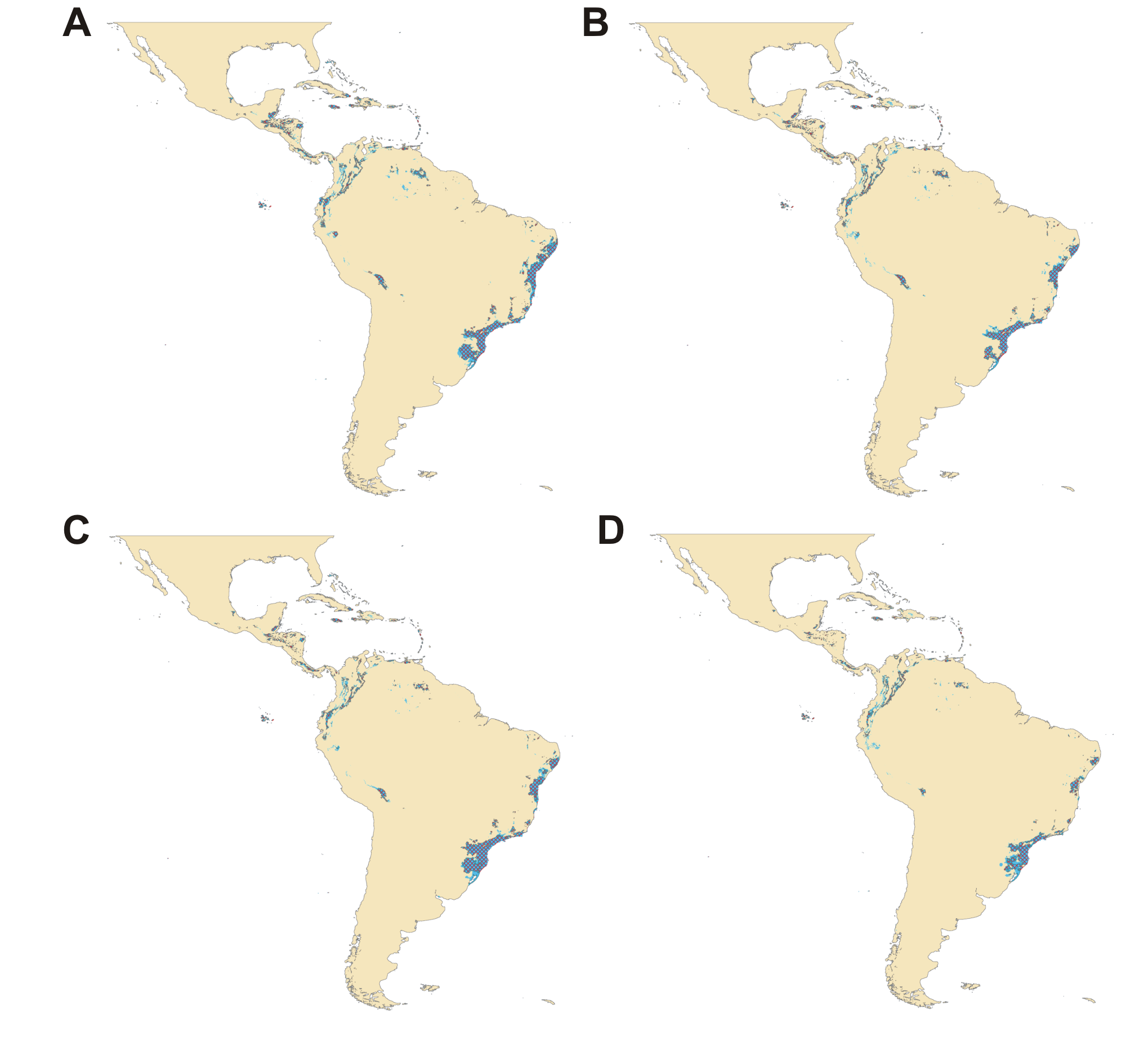


Fig. 3. Overlap of Asian binary models created using “fade-by-clamping” option in MaxEnt (red hatch) and without this option (blue filling) based on rcp2.6 (A), rcp4.5 (B), rcp6.0 (C), and rcp8.5 (D) scenarios.


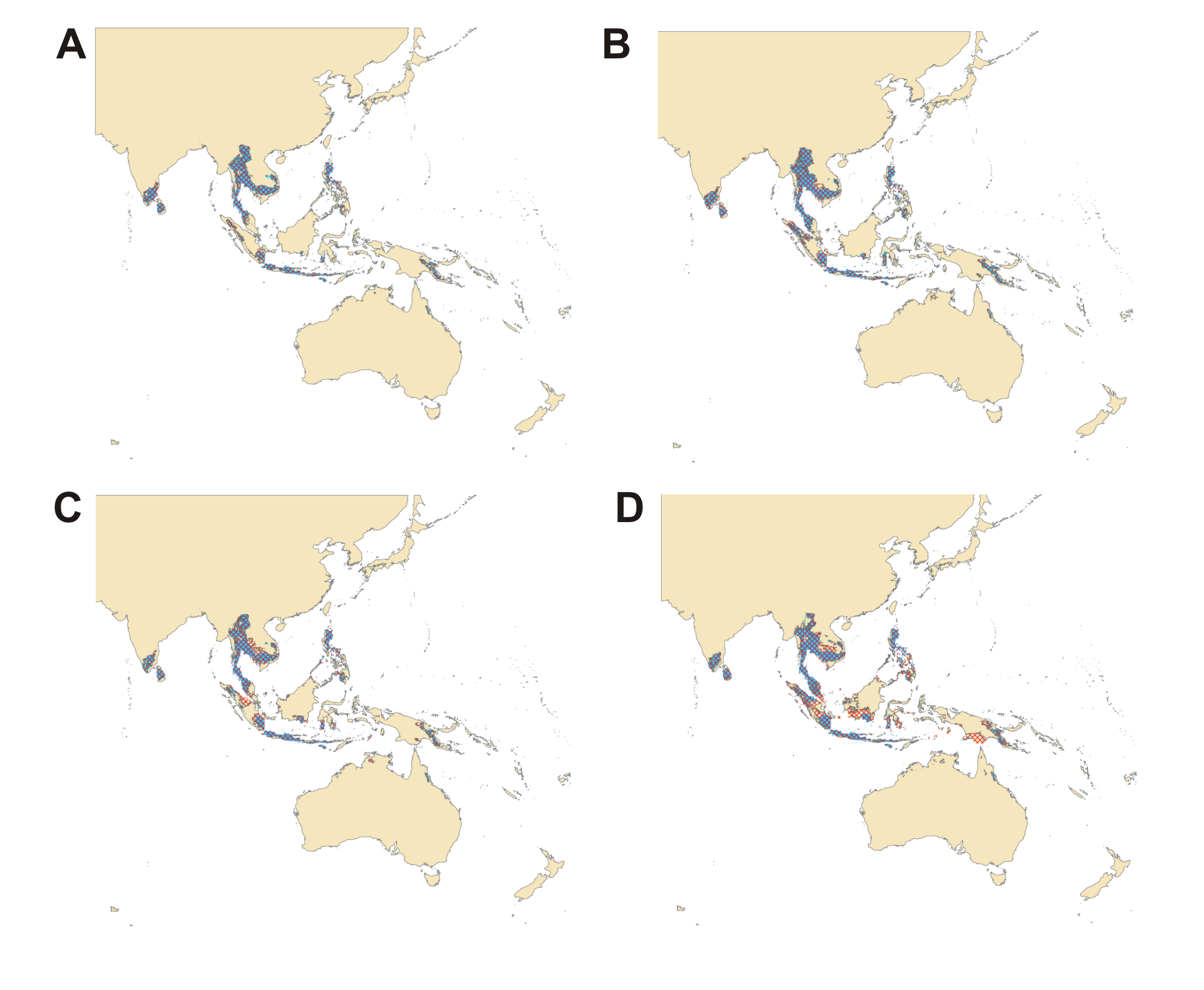


Fig. 4. Changes in the distribution of suitable niches of *P. concreta* in Africa based on rcp2.6 (A), rcp4.5 (B), rcp6.0 (C), and rcp8.5 (D) scenarios. SDMtoolbox output maps visualized in ArcGIS 10.6 (Esri, Redlands, CA, USA).


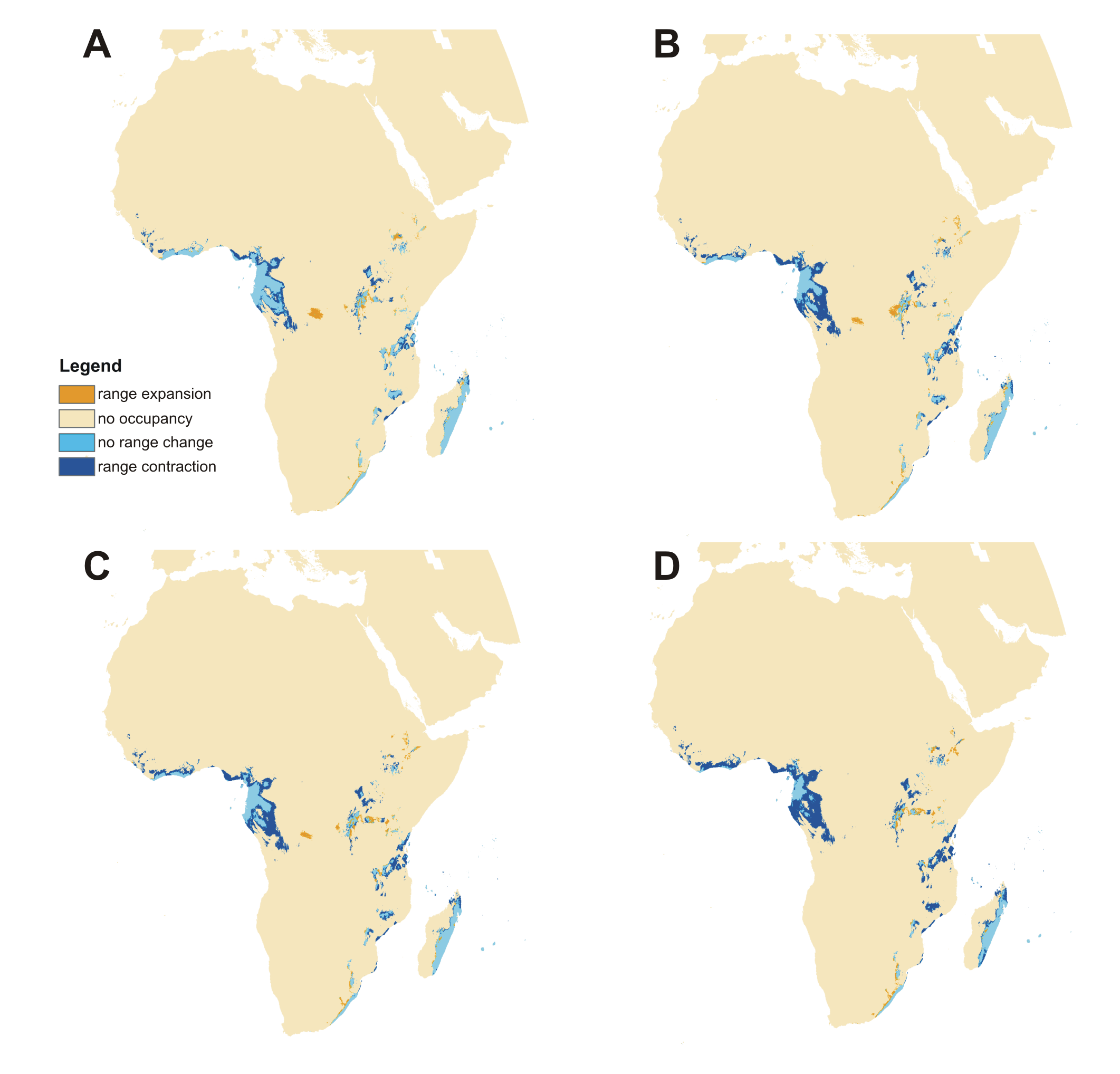


Fig. 5. Changes in the distribution of suitable niches of *P. concreta* in America based on rcp2.6 (A), rcp4.5 (B), rcp6.0 (C), and rcp8.5 (D) scenarios. SDMtoolbox output maps visualized in ArcGIS 10.6 (Esri, Redlands, CA, USA).


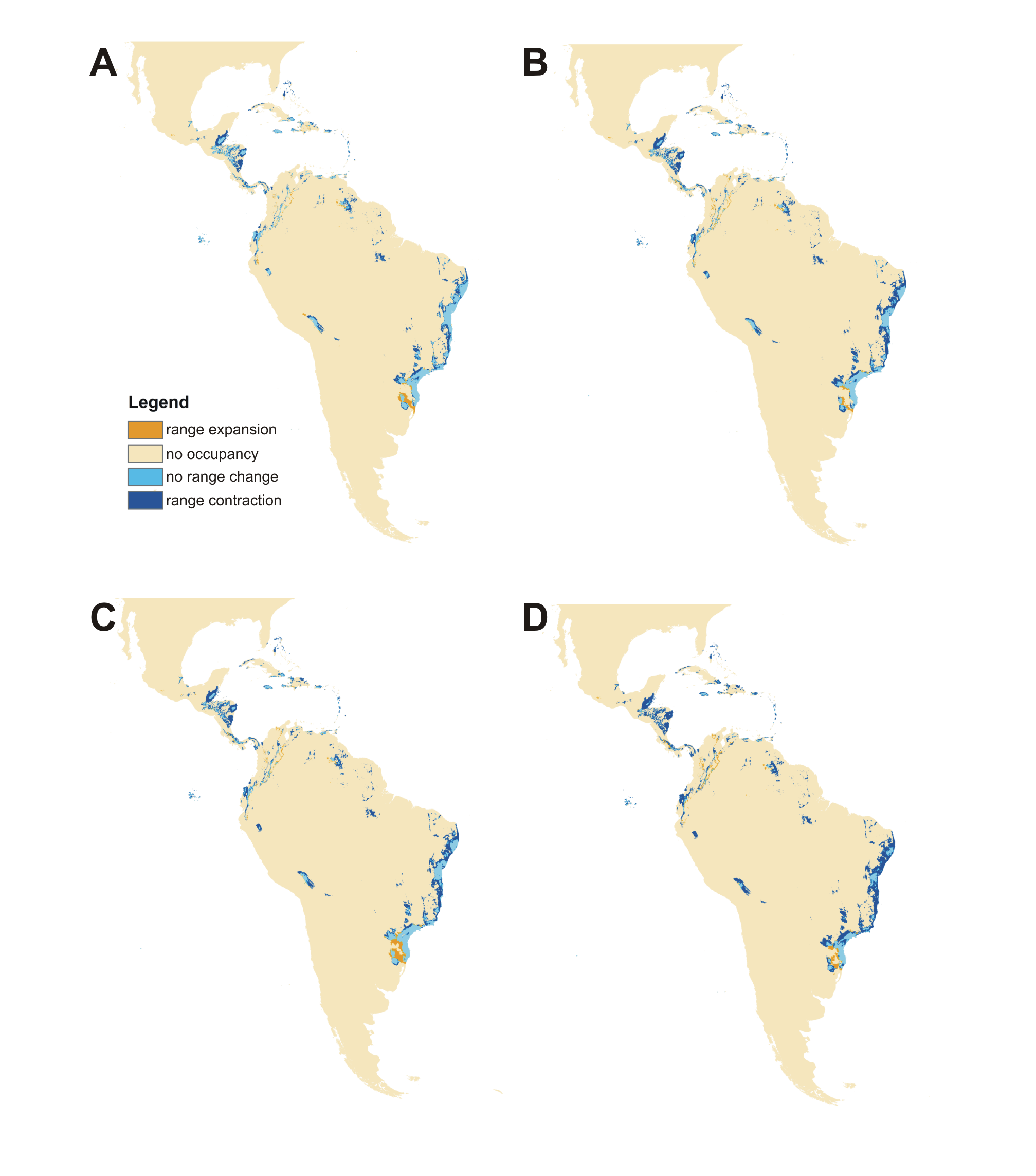


Fig. 6. Changes in the distribution of suitable niches of *P. concreta* in Asia based on rcp2.6 (A), rcp4.5 (B), rcp6.0 (C), and rcp8.5 (D) scenarios. SDMtoolbox output maps visualized in ArcGIS 10.6 (Esri, Redlands, CA, USA).


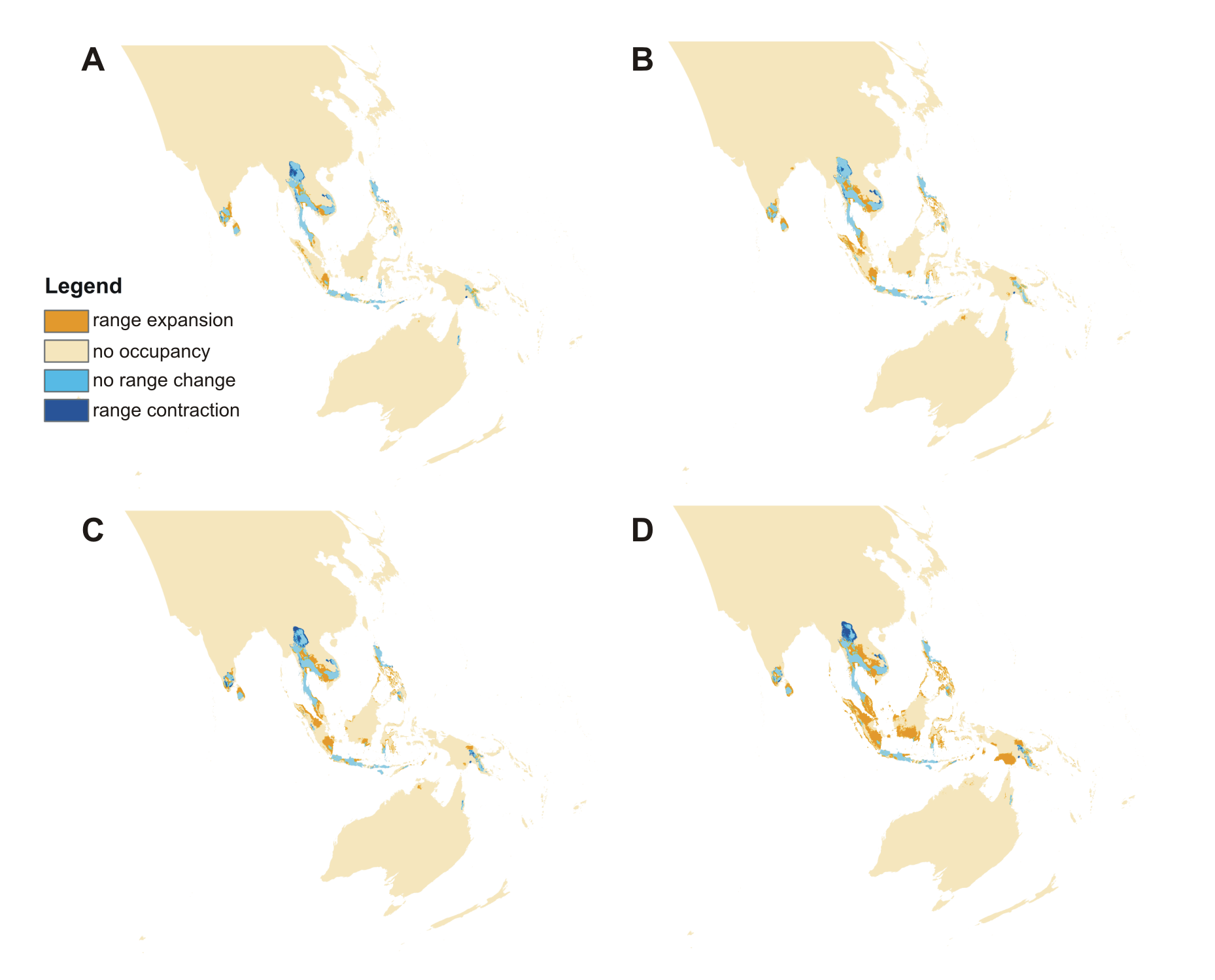


**Table 1**. Results of the TSS tests.

| Region | Present | rcp2.6 | rcp4.5 | rcp6.0 | rcp8.5 |
| --- | --- | --- | --- | --- | --- |
| America | 0.604055 | 0.6502753 | 0.612039 | 0.617539 | 0.5619072 |
| Africa | 0.7914507 | 0.8139507 | 0.8004258 | 0.8098005 | 0.8225502 |
| Asia | 0.9017 | 0.8866 | 0.8579 | 0.8568459 | 0.886873 |

**Table 2**. The average training AUC for the replicate runs for the created models.

| Region | Present | rcp2.6 | rcp4.5 | rcp6.0 | rcp8.5 |  |
| --- | --- | --- | --- | --- | --- | --- |
| America | 0.924 (SD=0.006) | 0.925 (SD=0.006) | 0.924 (SD=0.007) | 0.923 (SD=0.007) | 0.924 (SD=0.007) | |
| Africa | 0.960 (SD=0.003) | 0.960 (SD=0.003) | 0.961 (SD=0.003) | 0.961 (SD=0.003) | 0.959 (SD=0.003) | |
| Asia | 0.985 (SD=0.002) | 0.984 (SD=0.003) | 0.982 (SD=0.003) | 0.984 (SD=0.003) | 0.984 (SD=0.003) | |

**Table 3**. Estimates of relative contributions of the environmental variables to the Maxent models.

| Region | var. 1 | var. 2 | var. 3 |
| --- | --- | --- | --- |
| America | bio4 (19.1%) | bio2 (18.2%) | bio1 (14.6%) |
| Africa | bio12 (43%) | bio18 (17.0%) | bio14 (16%) |
| Asia | bio4 (36.3%) | bio3 (16.6%) | bio15 (10.2%) |

**Table 4**. Changes in the coverage of suitable niches of *P. concreta*.

| Region | Scenario | Range expansion [km^2^] | No occurrence [km^2^] | No change [km^2^] | Range contraction [km^2^] | Habitat loss / expansion |
| --- | --- | --- | --- | --- | --- | --- |
| Africa | rcp26 | 128528.52 | 35810142.34 | 876151.51 | 433133.93 | -23.27% |
|  | rcp45 | 130069.63 | 35808601.23 | 650368.71 | 658916.73 | -40.39% |
|  | rcp60 | 126638.10 | 35812032.77 | 627724.67 | 681560.76 | -42.38% |
|  | rcp85 | 103583.10 | 35835087.77 | 415298.16 | 893987.28 | -60.37% |
| America | rcp26 | 89077.77 | 21288258.23 | 614542.061 | 557107.38 | -39.95% |
|  | rcp45 | 81223.26 | 21296112.74 | 460512.70 | 711136.75 | -53.76% |
|  | rcp60 | 124501.82 | 21252834.18 | 470842.84 | 700806.61 | -49.19% |
|  | rcp85 | 97674.96 | 21279661.03 | 245290.19 | 926359.25 | -70.73% |
| Asia | rcp26 | 340915.93 | 31285270.78 | 881742.41 | 115622.54 | +22.59% |
|  | rcp45 | 672657.34 | 30953529.37 | 912159.49 | 85205.46 | +58.90% |
|  | rcp60 | 735985.09 | 30890201.62 | 840057.13 | 157307.81 | +58.02% |
|  | rcp85 | 1533533.59 | 30092653.12 | 826036.58 | 171328.37 | +136.58% |

**Table 5**. Niche overlap between models created using “fade-by-clamping” option and without this function.

| Region | Scenario | D | I |
| --- | --- | --- | --- |
| Africa | present | 0.93851 | 0.99586 |
|  | rcp26 | 0.92374 | 0.99409 |
|  | rcp45 | 0.91687 | 0.99301 |
|  | rcp60 | 0.92029 | 0.99217 |
|  | rcp85 | 0.83077 | 0.96955 |
| America | present | 0.88763 | 0.98979 |
|  | rcp26 | 0.88071 | 0.98728 |
|  | rcp45 | 0.86003 | 0.98377 |
|  | rcp60 | 0.86885 | 0.98441 |
|  | rcp85 | 0.86651 | 0.98190 |
| Asia | present | 0.93840 | 0.99634 |
|  | rcp26 | 0.92612 | 0.99456 |
|  | rcp45 | 0.90710 | 0.99081 |
|  | rcp60 | 0.90028 | 0.98892 |
|  | rcp85 | 0.86035 | 0.97506 |
